# Possible Role of Diffusion-Weighted Imaging in Prediction of Prostate Cancer Grade Group Upgrading: Insights from Biopsy to Radical Prostatectomy

**DOI:** 10.3390/medicina62040750

**Published:** 2026-04-14

**Authors:** Anna Żurowska, Katarzyna Skrobisz, Marek Sowa, Rafał Pęksa, Damian Panas, Małgorzata Grzywińska, Marcin Matuszewski, Edyta Szurowska

**Affiliations:** 1 Second Department of Radiology, Medical University of Gdansk, 80-214 Gdansk, Poland; 2 Department of Radiology, Medical University of Gdansk, 80-214 Gdansk, Poland; 3 Department of Urology, Medical University of Gdansk, 80-214 Gdansk, Poland; 4 Department of Pathomorphology, Medical University of Gdansk, 80-214 Gdansk, Poland; 5 Molecular Biology Laboratory, Institute of Animal Reproduction and Food Research Polish Academy of Sciences, 10-748 Olsztyn, Poland; 6 Neuroinformatics and Artificial Intelligence Lab, Department of Neurophysiology, Neuropsychology and Neuroinformatics, Medical University of Gdansk, 80-210 Gdansk, Poland

**Keywords:** prostate cancer (PCa), magnetic resonance imaging (MRI), diffusion weighted imaging (DWI), diffusion kurtosis imaging (DKI), Gleason score (GS), Gleason Grade Group (GG)

## Abstract

*Background and Objectives*: Prostate cancer is the second most common cancer in men worldwide, with 1,466,680 new cases and 396,792 deaths reported in 2022. Accurate preoperative grading is critical, as the grade assessed on biopsy cores may be underestimated compared to radical prostatectomy specimens. The aim of this study was to assess the ability of quantitative diffusion parameters derived by the standard monoexponential model (ADC—apparent diffusion coefficient) and kurtosis model (Dapp—apparent diffusion coefficient corrected for non-Gausion behavior and K-kurtosis) to predict Gleason Grade Group (GG) upgrading from transrectal ultrasound-guided (TRUS) biopsy to radical prostatectomy within each GG. *Materials and Methods*: This retrospective study included 128 patients with prostate cancer who underwent systematic TRUS biopsies and multiparametric magnetic resonance imaging (mpMRI) at 3T before prostatectomies between 2017 and 2021. Mean values of quantitative diffusion parameters (ADC, Dapp, K) were compared between upgraded and non-upgraded cohorts within each Grade Group obtained at biopsy. *Results*: Significant differences in ADC and K values were found between upgraded and non-upgraded lesions in GG1 and GG2 cohorts at biopsy, with lower ADCs and higher K values indicating a higher likelihood of upgrading. In GG1, ADC demonstrated an AUC of 0.762 (*p* < 0.05) and K an AUC of 0.846 (*p* < 0.05). In GG2, ADC showed an AUC of 0.814 (*p* < 0.001) and K an AUC of 0.755 (*p* < 0.001). No significant differences were observed in GG3 and GG4 cohorts. *Conclusions*: Quantitative diffusion parameters—particularly ADC and kurtosis (K)—demonstrated significant predictive value for Grade Group upgrading in patients with biopsy-proven GG1 (AUC: K = 0.846, ADC = 0.762) and GG2 (AUC: ADC = 0.814, K = 0.755, D = 0.810) prostate cancer. These findings suggest that incorporating quantitative DWI parameters into preoperative assessments may improve risk stratification and support clinical decision-making, particularly regarding the selection of patients for active surveillance. Validation in larger, multicenter cohorts is warranted.

## 1. Introduction

Cancer represents a major global public health challenge. In the United States alone, approximately 2,114,850 new cancer cases and 626,140 cancer deaths are projected to occur in 2026 [1]. Among malignancies affecting men, prostate cancer is of particular significance: it is the second most common cancer in men worldwide, with 1,466,680 new cases and 396,792 deaths reported in 2022 [2]. Multiple biological and molecular factors are implicated in the progression of prostate cancer, including androgen receptor signaling pathways and innate immune mechanisms such as Toll-like receptor (TLR) signaling [3].

A definite diagnosis of prostate cancer is based on the finding of adenocarcinoma in prostate biopsy cores [4]. Therefore, accurate assessment of the grade of prostate cancer (PCa) according to Gleason score (GS) and the International Society of Urological Pathology (ISUP) Grade Groups (GG) [5,6] is crucial because it constitutes the basis for implementing appropriate treatment, ranging from active surveillance for selected low-risk cancers to radical treatment of significant PCa [4].

However, systematic transrectal ultrasound-guided (TRUS) biopsies sample a limited portion of the prostate gland, and as a consequence, there is a substantial risk of both the undergrading and overgrading of prostate cancer compared to GG assessed after radical prostatectomy [7,8,9,10,11,12].

The development of multiparametric MRI (mpMRI) examinations and their use prior to biopsy in patients with suspected prostate cancer has enabled the introduction of fusion biopsies. Three basic types of fusion biopsy can be distinguished: cognitive biopsy, which involves visual correlation of MRI and TRUS images by the physician performing the biopsy; software-assisted fusion of MRI and TRUS real-time images; and MRI-guided in-bore biopsy.

The introduction of mpMRI and fusion biopsies has led to an increase in the detection of clinically significant prostate cancers, a decrease in the detection of low-grade prostate cancers (GG1), and a reduction in the rate of prostate cancer Grade Group upgrades from biopsy to post-radical prostatectomy examination [13,14].

Unfortunately, even with targeted fusion biopsies, some upgrades in the Grade Group of prostate cancer obtained from biopsy compared to post-radical prostatectomy examinations are still observed [13,14,15,16,17,18].

As a consequence, upgraded pathology after radical prostatectomy (RP) can have serious clinical implications, as men with clinically significant prostate cancer may be undertreated.

In recent years, mpMRI has evolved into a pivotal diagnostic imaging modality in the diagnosis of prostate cancer. In particular, the quantitative parameters of diffusion-weighted imaging (DWI) have gained interest as non-invasive markers for diagnosing and predicting the grading of prostate cancer [19,20,21,22].

Several studies have shown that quantitative ADC might be helpful in the prediction of upgrading of GS in TRUS-guided biopsy-proven low-grade prostate cancer (GS of 6) [23,24]. Furthermore, Wu et al. revealed that diffusion kurtosis imaging might be helpful in the prediction of the upgrade of biopsy-proven PCa with a GS of 6 compared to radical prostatectomy [25].

A few studies have evaluated the role of quantitative ADC (including ADC histogram and texture analysis) to predict GS upgrade in intermediate-risk (GS 3 + 4) prostate cancer [26].

The diagnostic performance of diffusion kurtosis imaging in comparison to the standard monoexponential diffusion model (ADC) in the diagnosis of prostate cancer has already been evaluated in several studies [27,28,29] showing high concordance between these two diffusion models, with no significant differences between them. However only a few studies have evaluated the role of DKI in predicting an upgrade in Gleason score 6 (3 + 3) of PCa diagnosed at biopsy compared to radical prostatectomy [25]. According to our knowledge, no studies have evaluated the role of diffusion kurtosis imaging (DKI) in predicting an upgrade in GS 3 + 4 prostate cancer diagnosed at biopsy.

Standard calculations of the apparent diffusion coefficient (ADC) are performed with a monoexponential model (ME), which assumes a Gaussian diffusion of water molecules and a linear decay of the logarithmic diffusion signal intensity as the b-value increases [30,31]. However, when b-values exceed 1000 s/mm^2^, a growing proportion of molecules encounter cellular membranes and other molecules, leading to deviations from free Gaussian distribution and loss of linearity in the logarithmic decay plot [30,32,33].

Diffusion kurtosis imaging (DKI) is a more complex diffusion model that accounts for the non-Gaussian behavior of water molecules at ultrahigh b values. DKI provides two variables: kurtosis (K), a dimensionless measure of the extent of non-Gaussian diffusion, and Dapp, the apparent diffusion coefficient corrected for non-Gaussian behavior (expressed in units: 10^−3^ mm^2^/s) [34].

DKI has the potential to add additional microstructural information about prostate cancer, which is known for its histological heterogeneity, encompassing a mix of higher- and lower-grade components within a single lesion [5]. It is hypothesized that DKI could enhance the differentiation of different tissue types [28].

Our study aimed to evaluate quantitative DWI parameters derived by the standard monoexponential model (ADC) and kurtosis model (Dapp and K) in the prediction of GG upgrading from TRUS-guided biopsy to radical prostatectomy within each ISUP Grade Group.

## 2. Materials and Methods

### 2.1. Study Population

This retrospective single-institution study included 128 patients with a mean Prostate-Specific Antigen (PSA) level of 9.67 ng/mL (range 2.2–28.5 ng/mL), with prostate cancer who had undergone systematic TRUS biopsy and mpMRI at 3T before prostatectomy (performed between 2017 and 2021). The inclusion criteria were: (1) histopathologically confirmed prostate cancer on TRUS-guided systematic biopsy; (2) mpMRI at 3T performed before radical prostatectomy; (3) radical prostatectomy performed at our institution with whole-mount histopathological assessment. The exclusion criteria were: (1) prior neoadjuvant therapy, as this could affect diffusion parameter measurements; (2) poor mpMRI image quality precluding reliable quantitative diffusion analysis.

The study was approved by the Institutional Ethics Committee of the Medical University of Gdansk (approval number: NKBBN/126/2018). The requirement for informed consent was waived due to the retrospective nature of the study.

### 2.2. Data Acquisition

All MRI examinations were conducted using a Philips Achieva 3.0 T Tx MRI scanner (Philips Achieva 3.0 T Tx, Best, Netherlands) equipped with a 32-channel cardiac coil. The diffusion-weighted imaging (DWI) protocol utilized a single-shot echo-planar imaging sequence in the axial plane, with a repetition time (TR) of 2000 ms and an echo time (TE) of 70 ms. The field of view (FOV) was set to 180 × 250 mm, with a matrix size of 80 × 112, a slice thickness of 3.5 mm, and an inter-slice gap of 0 to 0.35 mm. The DWI protocol included six b-values (0, 100, 500, 800, 1200, and 2000 s/mm^2^) with five signal averages for each b-value. Additionally, the prostate multiparametric MRI (mpMRI) protocol included T2-weighted imaging in three planes, T1-weighted imaging, and dynamic contrast-enhanced (DCE) imaging.

### 2.3. Data Analysis

DICOM images were transferred from the Picture Archiving and Communication System (PACS) to specialized software (Intellispace Portal 10, Advanced Diffusion Analysis application). This software was employed to generate maps of the apparent diffusion coefficient (Dapp) and kurtosis (K) using the kurtosis model equation:

S(b) = S(0) exp (−b × D + b2 × Dapp2 × K/6)

using all b values, and ADC with the standard monoexponential model:

S(b) = S(0) exp (−bADC)

using b values up to 1200 s/mm^2^.

The preprocessing steps in the Advanced Diffusion Analysis application included motion correction and eddy current correction.

All examinations were reviewed by two radiologists with 4 and 13 years of experience in prostate MRI. By consensus, they identified the location and extent of the prostate cancer (PCa) index lesion(s), correlating these findings with the histopathologic results of the prostatectomy specimens. A PI-RADS (Prostate Imaging—Reporting and Data System) score was assigned according to PI-RADS version 2.1 [35].

Quantitative diffusion parameters were obtained by the radiologist with 4 years of experience. Regions of interest (ROIs) were drawn on each patient’s dominant lesion(s) on the generated apparent diffusion coefficient (ADC) maps. Subsequently these ROIs were automatically copied to the D and K maps, and the quantitative values of each parameter were calculated. The ROI in the PCa foci was drawn within the lesion, excluding the margins, on the scan with the lowest ADC to avoid partial volume effects.

### 2.4. Histopathology

Patients had undergone TRUS-guided systematic biopsy with 12 cores before prostatectomy. The biopsy was performed before or after mpMRI. The median time from mpMRI to biopsy or from biopsy to mpMRI was 5.5 weeks (range 4–10 weeks). The median time from mpMRI do prostatectomy was 6.5 weeks (range 2–10 weeks).

After radical prostatectomy, the resected prostate was inked and fixed in formalin. The specimen was then processed using whole-mount techniques, involving slicing into 4 mm sections perpendicular to the urethra. Each section was subsequently stained with hematoxylin and eosin.

The Gleason score (GS) of tumors according to the 4th Edition of the WHO Classification of Tumors of the Urinary System and Male Genital Organs [36] and ISUP Grade Groups [37] were used to assess the specimens.

The upgrade was defined as an increase from a lower to a higher ISUP Grade Group from biopsy to radical prostatectomy specimen.

Lesions within each Grade Group obtained by biopsy were divided into lesions that presented an upgrade at the final histopathology after radical prostatectomy and lesions without an upgrade. Quantitative diffusion parameters were compared between the upgraded and non-upgraded cohorts within each Grade Group of prostate cancer obtained by biopsy. The statistical analysis was performed on a per-lesion basis. Each lesion was treated as an independent observation with its own set of quantitative diffusion parameters.

### 2.5. Statistical Analysis

Statistical analysis was performed using R 4.2.2. To investigate the diagnostic ability of selected diffusion parameters, logistic regression with 5 repeats of 5-fold cross-validation as a resampling method was implemented. For each model obtained, a receiver operating characteristic (ROC) was plotted. Additionally, specificity, sensitivity, and area under the curve (AUC) were calculated. To find the optimal cut-off value, the Youden index was applied. To compare levels of diffusion parameters between groups independent samples *t*-test or Mann–Whitney U-test was employed. In addition, Cohen’s d statistic was calculated to measure effect size. In the case of multiple comparisons, the Holm correction method was applied. Throughout the study, a 5% significance level maintained.

## 3. Results

A total of 142 tumors were studied. Overall, 105 (74%) were located in the peripheral zone and 37 (26%) in the transition zone at mpMRI and whole-mount histopathology specimens after prostatectomies. According to PI-RADS version 2.1 [35], four lesions were classified as PI-RADS 3, 71 lesions as PI-RADS 4, and 67 lesions as PI-RADS 5.

In total 68 lesions had GG upgrades (68/142; 47.9%) from biopsy to radical prostatectomy. In the upgrade cohort 20 lesions were upgraded from GG 1 on biopsy to higher GG after radical prostatectomy (20/68; 29.4%), 36 lesions were upgraded from GG 2 (36/68; 52.9%), eight lesions were upgraded from GG 3 (8/68; 11.8%), four lesions from GG 4 (4/68; 5.9%).

Within each ISUP Grade Groups in biopsy, the upgrade cohort was as follows: in GG 1 in biopsy: 20 lesions out of 27 were upgraded (20/27; 74.07%), in GG 2: 36 lesions out of 67; (36/67; 53.73%), in GG 3: 8 lesions out of 35 (8/35; 22.86%), in GG 4: 4 lesions out of 13 (4/13: 30.77%). Characteristics of study population, PI-RADS score and ISUP Grade Groups obtained in preoperative biopsy and at final pathology after prostatectomy are listed in Table 1.

### 3.1. Grade Group 1 in Biopsy

In patients with ISUP Grade Group 1 in biopsy, 20 lesions out of 27 were upgraded to higher Grade Groups (20/27; 74.07%).

Mean values of K and ADC in tumors between the upgraded and non-upgraded cohorts were significantly different. In the upgraded cohort, mean ADC values in cancerous foci were significantly lower (0.761 vs. 0.891 × 10^−3^ s/mm^2^) and mean K values were significantly higher (1.262 vs. 1.086) in comparison to the cohort without an upgrade. Mean values of D between the upgraded and non-upgraded groups were not statistically significant (*p* > 0.05).

**Figure 1:**
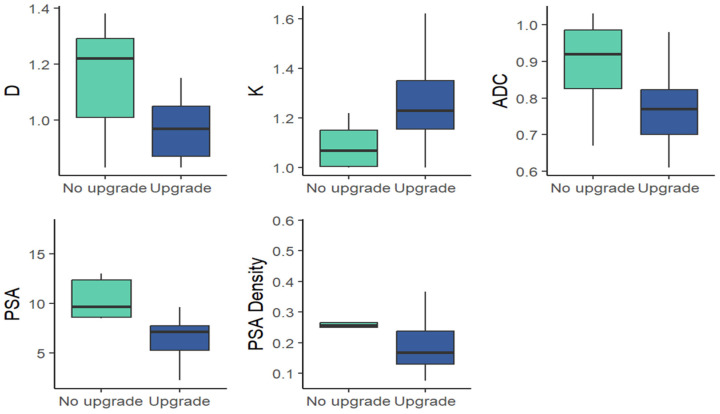
Box-and-whisker plots of mean values for DKI parameters (D, K), standard ADC and clinical parameters (PSA, PSAD) in the upgrade and non-upgrade cohorts within GG 1 at biopsy.

Preoperative clinical variables (PSA, PSA density) were not significantly different between the two cohorts Figure 1 and Table 2.

**Figure 2:**
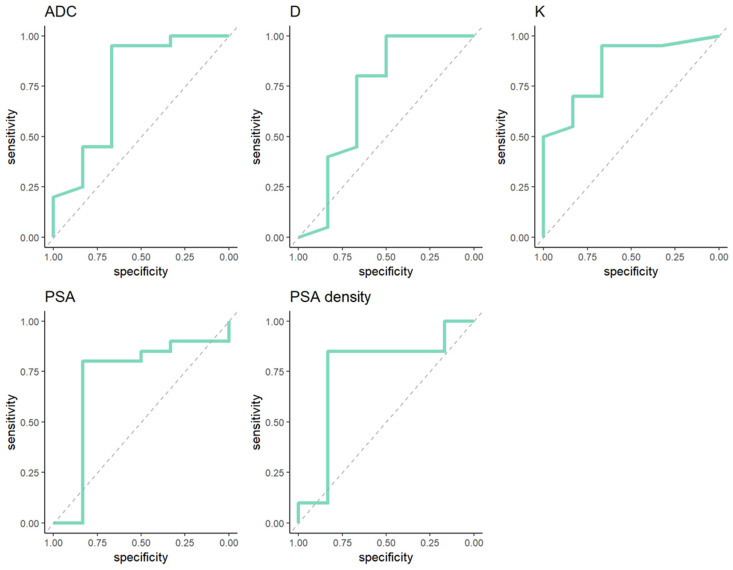
ROC analysis curves with diagnostic performance of diffusion parameters and clinical variables in prediction of GG upgrade from biopsy to radical prostatectomy within GG 1 at biopsy.

ROC analysis curves with diagnostic performance of diffusion parameters (ADC, D, K) and clinical variables (PSA, PSA density) in prediction of GG upgrade from biopsy to radical prostatectomy are demonstrated in Figure 2.

Youden index thresholds, AUC, sensitivity, specificity for each variable are depicted in Table 3.

Among diffusion parameters kurtosis demonstrated the highest diagnostic performance in prediction of GG upgrade from biopsy to radical prostatectomy, with an AUC of 0.846, a sensitivity of 0.667 and a specificity of 0.95 respectively.

### 3.2. Grade Group 2 in Biopsy

In patients with ISUP Grade Group 2 in biopsy, 36 lesions out of 67 were upgraded from GG 2 to a higher GG from biopsy to radical prostatectomy (36/67; 53.73%).

In this group, mean values of all diffusion parameters (K, D and ADC) in tumors were significantly different between the upgraded and non-upgraded cohorts (*p* < 0.001). In the upgraded cohort, mean ADC and D values were significantly lower and mean K values were significantly higher.

**Figure 3:**
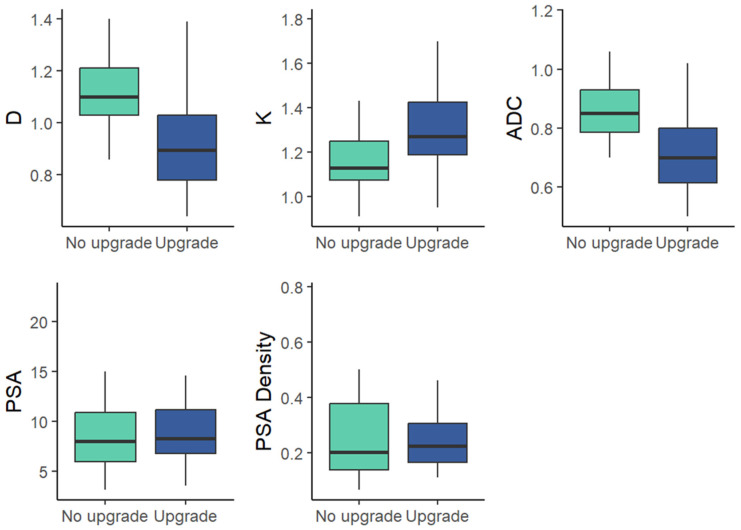
Box-and-whisker plots of mean values for DKI parameters and clinical parameters in the upgrade and non-upgrade cohorts within GG 2 at biopsy.

Preoperative clinical variables (PSA, PSA density) were not significantly different between the two cohorts (*p* > 0.05) Figure 3 and Table 4.

**Figure 4:**
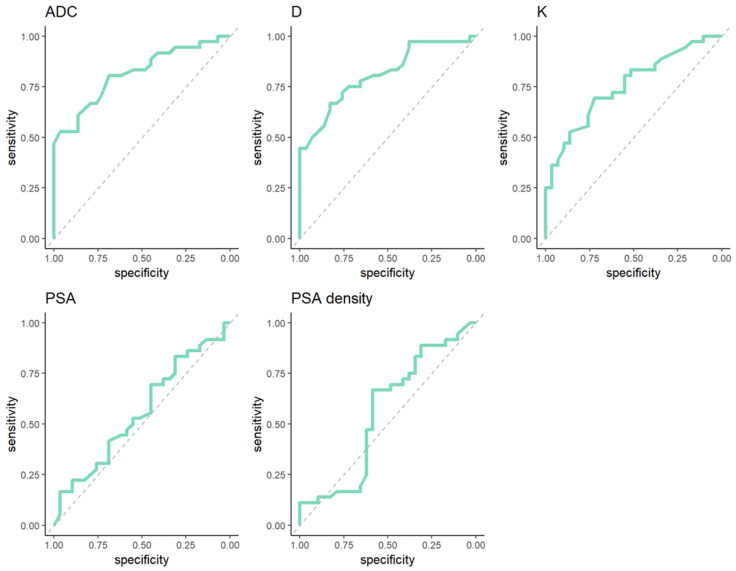
ROC analysis curves with diagnostic performance of diffusion parameters and clinical variables in prediction of GG upgrade from biopsy to radical prostatectomy within GG 2 at biopsy.

Diagnostic performance of diffusion parameters (ADC, D, K) and clinical variables (PSA, PSA density) by ROC analysis in prediction of GG upgrade from biopsy to radical prostatectomy in patients with ISUP GG 2 diagnosed in preoperative biopsy are demonstrated in Figure 4.

Optimal thresholds values for diffusion parameters (ADC, D, K) and clinical variables (PSA, PSA density) by Youden index in the prediction of GG upgrade and the respective sensitivity, specificity and calculated AUC are depicted in Table 5.

Diagnostic performance of all diffusion parameters (K, D, ADC) was similar by ROC analysis with an AUC that ranged from 0.755 for K to 0.814 for ADC. The AUCs for diagnostic performance of PSA and PSA density were 0.561 and 0.557 respectively.

### 3.3. Grade Group 3 and 4 in Biopsy

In patients with ISUP Grade Group 3 in biopsy, 8 lesions out of 35 were upgraded from GG 3 to higher GG from biopsy to radical prostatectomy (8/35; 22.86%).

In patients with ISUP Grade Group 4 in biopsy, 4 lesions out of 13 were upgraded from GG 4 to GG 5 from biopsy to radical prostatectomy (4/13: 30.77%).

In these groups mean values of ADC, D and K in the upgraded cohort compared to cohort without an upgrade, were not statistically significant.

The mean values of PSA and PSA density also showed no significant differences between the cohorts (*p* > 0.05).

**Figure 5:**
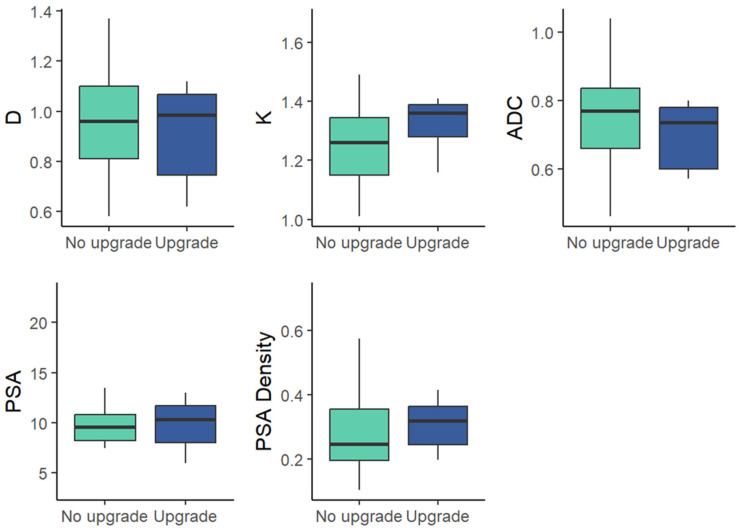
Box-and-whisker plots of mean values for DKI parameters and clinical parameters in the upgrade and non-upgrade cohorts within GG 3 at biopsy.

**Figure 6:**
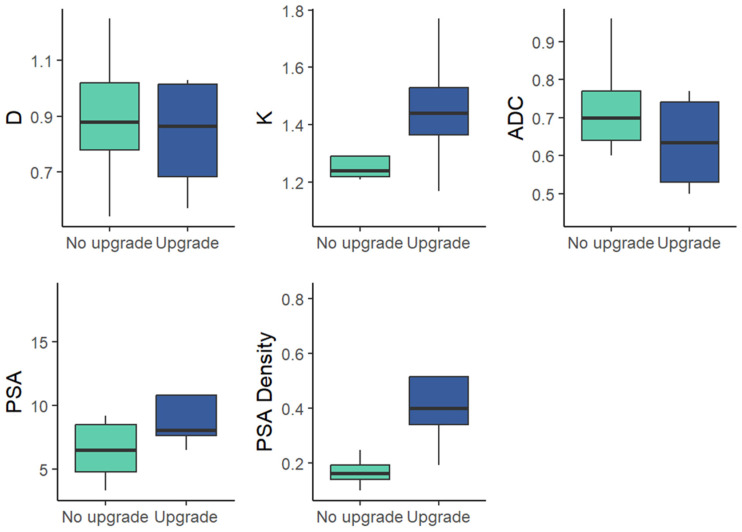
Box-and-whisker plots of mean values for diffusion parameters and clinical parameters in the upgrade and non-upgrade cohorts within GG 4 at biopsy.

The mean values of diffusion parameters (K, D, ADC) and clinical variables (PSA, PSA density) in differentiation between upgraded cohort and the cohort without upgrade in the patients with ISUP GG 3 and 4 in preoperative biopsy are demonstrated in Figure 5, Table 6 and Figure 6, Table 7 respectively.

## 4. Discussion

The results of our study revealed that the mean values of quantitative diffusion parameters derived both by the standard monoexponential diffusion model (ADC) and by the kurtosis model (K) were able to predict the ISUP Grade Group upgrade among patients diagnosed with GG 1 and GG 2 prostate cancer at biopsy, except for Dapp in GG 1 at biopsy (*p* 0.063).

However, in both DKI and ADC parameters an overlap of values was observed between the upgrade and non-upgrade cohorts. The mean values of diffusion parameters in patients diagnosed with GG 3 and GG 4 prostate cancer at biopsy, were not statistically significant between the upgraded cohort and cohort without an upgrade.

On a microstructural level, diffusion weighted imaging has the capacity to reflect the Brownian motion of water molecules within human tissues, thereby providing indirect information regarding tumor cellularity and the structural integrity of cell membranes [30,34].

Tumors are characterized by significantly increased cellularity, as well as decreased fibromuscular stroma matrix and decreased luminal space compared to normal tissue [38]. Higher grade prostate cancer tumors (ISUP GG ≥ 3) have significantly increased cellularity and decreased fibromuscular stroma compared to ISUP GG ≤ 2 tumors [38]. The inverse correlation between ADC and Dapp values and ISUP GG that means lower ADC and Dapp are observed in higher grade tumors. On the contrary, mean kurtosis values are positively correlated with ISUP GG, meaning the higher kurtosis values are observed in higher grade tumors.

It was demonstrated in previous studies that ADC values correlate with cell density [39] and cell proliferation rate assessed as Ki-67 [40] as well as with prostate cancer histopathological grade assessed by Gleason score [19,20,21,22].

What is more, Lawrence E.M. et al. showed the positive correlation between median kurtosis (Kapp) and cellularity as well as tissue heterogeneity [38]. Yin H. et al. demonstrated correlation between mean kurtosis and Gleason score [41].

The overlap of DKI and ADC parameters between the upgrade and non-upgrade cohorts may be explained by the histologically heterogeneous nature of prostate cancer, that often is composed of a mixture of higher- and lower-grade components within a single lesion on a microscopic level. Moreover, even though diffusion weighted imaging reflects processes occurring at the microscopic level, it has a certain spatial resolution and in our study it was assessed on a macroscopic level. Future studies exploring more advanced analysis including radiomics, texture analysis and machine learning are needed.

Several studies have already demonstrated that quantitative ADC might be helpful in the prediction of the upgrading of GS in biopsy-proven low-grade prostate cancer (GS of 6) [23,24]. Furthermore, Wu et al. revealed that diffusion kurtosis imaging might be helpful in the prediction of the upgrade in biopsy-proven PCa with a GS of 6 compared to radical prostatectomy [25]. Some studies have evaluated the role of quantitative ADC (including ADC histogram and texture analysis) to predict a GS upgrade in intermediate-risk (GS 3 + 4) prostate cancer [26]. According to our knowledge, no studies have evaluated the role of diffusion kurtosis imaging (DKI) in predicting an upgrade in GS 3 + 4 prostate cancer diagnosed at biopsy.

Our findings are consistent with the previous studies of Somford et al. [23] and Park et al. [24]. Similarly to previous studies, we have shown that mean values of quantitative ADC can be helpful in preoperative evaluation of the upgrade of ISUP GG 1 obtained at TRUS biopsy (*p* < 0.05). The diagnostic performance of the ADC in the upgrade of GG 1 prostate cancer in ROC analysis in our study, with an AUC of 0.762, was comparable with the diagnostic performance of ADC in Park et al. [24] with an AUC of 0.760 for ADC min and an AUC of 0.711 for ADC mean; our study is also comparable to that of Somford, with an AUC of 0.88 for median ADC [23].

Our results demonstrated that a kurtosis parameter might also be useful in predicting the upgrade of GG1 PCa diagnosed at biopsy, with a diagnostic performance of K—AUC 0.846 in ROC analysis (*p* 0.05), which was similar to Wu et al.’s [25] findings with an ROC AUC of 0.819 (*p* < 0.05).

Recent studies have further corroborated the value of mpMRI in predicting histopathological upgrading. Boschheidgen et al. [42] demonstrated in a 2024 study that MRI characteristics, including ADC values and PI-RADS category, were significant predictors of pathological upgrades in patients with ISUP GG1 prostate cancer. Their findings showed that 55% of patients with an initial GG1 diagnosis experienced upgrading during follow-up, with ADC values being significantly different between upgraded and sustained GG1 cohorts. This is consistent with our observation of ADC’s predictive value in the GG1 cohort.

Similarly, Dekalo et al. [43] reported in 2024 that PI-RADS 4–5 lesions combined with ISUP 1 on biopsy were strong independent predictors of upgrading at radical prostatectomy (OR 24.3, 95% CI 7.3–80.5, *p* < 0.001), with 84% of patients with high PI-RADS scores experiencing upgrading compared to only 26% of those with equivocal or non-suspicious mpMRI findings. These findings underscore the importance of incorporating mpMRI and PI-RADS score into clinical decision-making algorithms.

Most recently, Zhang et al. [44] in a 2025 Korean Journal of Radiology publication evaluated multiparametric MRI to predict both Gleason score upgrading and downgrading at radical prostatectomy. Their study emphasizes the comprehensive role of mpMRI and supports our findings regarding the utility of incorporating mpMRI findings in preoperative assessment.

Moreover, Sattini et al. [18] in a recent 2025 radiology study of 780 lesions evaluated concordance between biopsy and surgical Grade Groups following in-bore MRI-targeted biopsies. Their findings demonstrated a substantial risk of undergrading, particularly in Grade Group 1, with 66.1% of bGG 1 lesions upgraded to sGG 2 or higher at radical prostatectomy, compared to only 16.0% upgraded in bGG 2–4 lesions (*p* < 0.001). The moderate to substantial concordance (weighted κ = 0.61) between biopsy and surgical grades emphasizes that even with advanced MRI-targeted biopsy techniques, significant upgrading persists, particularly in lower-grade cancers. These findings support our observation that GG1 cancers at biopsy carry a substantial risk of harboring higher-grade disease, reinforcing the clinical value of quantitative diffusion parameters in preoperative risk assessment.

Additionally, we demonstrated that mean ADC and DKI parameter values may predict a Grade Group upgrade among patients diagnosed with ISUP GG 2 prostate cancer at TRUS biopsy (*p* < 0.001). The AUC in ROC analysis for ADC, K and D were 0.814, 0.755 and 0.810 respectively (<0.001). This finding contrasts with the previous work of Rozenberg et al., which did not show such a correlation regarding to quantitative ADC (including ADC histogram and texture analysis) [26].

The differences may be attributed to the methods used for calculating quantitative parameters and differences in study populations. In our study, 53.73% of tumors (36 out of 67 lesions) were upgraded from ISUP GG 2 to higher GG (mostly GG 3, but also several to GG 4 and 5). Hence the upgraded cohort consisted of a mixture of higher-grade tumors, in contrast to previous studies in which upgraded groups consisted mostly of ISUP GG 3 lesions. In the presented study, an ROI was drawn in the PCa focus excluding tumor margins on the scan with the lowest ADC, which avoids the partial volume effect, whereas in previous works the whole lesion analysis was performed.

Current guidelines [4,45] allow considering active surveillance (AS) for selected men with favorable intermediate risks of Grade Group 2 prostate cancer [46]. Prostate MRI already plays an important role both in diagnosing patients with prostate cancer and monitoring during AS. However, the decision to recommend AS must be made with caution, particularly in patients diagnosed with GG1 at biopsy and that have lesions that are visible on mpMRI, especially those with low ADC values and PI-RADS scores of 4 and 5, given the substantial risk of harboring a higher-grade disease at final pathology. In our cohort, 74.07% of GG1 lesions at biopsy were upgraded at radical prostatectomy—a finding consistent with recent data from Sattini et al. [18], who reported upgrading in 66.1% of bGG1 lesions. Our results suggest that quantitative diffusion parameters—easily obtainable from standard mpMRI already performed in the diagnostic workup—may add valuable prognostic information to support shared decision-making in this clinically challenging group of patients.

Our work has demonstrated that adding quantitative diffusion parameters can help upgrade patients with prostate cancer from GG 1 and GG 2 obtained by TRUS-guided systematic biopsy.

As a result, these methods may be helpful in the more precise classification of patients into risk groups and selecting optimal treatments. Quantitative measurements of diffusion parameters obtained in suspicious lesions on mpMRI before fusion biopsy could potentially assist in selecting target lesions for targeted fusion biopsies and better assessing patients at risk of an upgrade in ISUP GG after radical prostatectomy.

Ozbozduman K. et al. demonstrated that minimum ADC (ADC min) and tumor size obtained on mpMRI before MRI-guided in-bore biopsy might be useful markers for assessing the risk of prostate cancer upgrade at final histopathology after RP [13].

In our study, we compared ISUP Grade Groups obtained from TRUS systematic biopsy with GGs after prostatectomy and correlated the results with quantitative diffusion parameters derived from mpMRI. It is known that TRUS systematic biopsy often results in both undersampling and undergrading. Nevertheless, it seems valuable to compare diffusion parameters within different Grade Groups obtained from TRUS systematic biopsy versus GGs after radical prostatectomy.

Our work also assessed clinical variables as PSA and PSA density in predicting ISUP Grade Group upgrade; however neither of these parameters were able to predict upgrades among any ISUP GG diagnosed at biopsy.

These findings are consistent with some studies [13] and inconsistent with other studies [25,47]. What is more, in our study PSA level and PSA density were lower in the upgraded cohort in patients with ISUP Grade Group 1 diagnosed at biopsy, which is probably due to small sample size of patients in this group and sample-specific characteristics.

Further studies including novel clinical biomarkers such as the Prostate Health Index (PHI) [48] in addition to quantitative diffusion parameters, as well as incorporating machine learning techniques [13,49] and radiomics [50,51], are required to further improve the preoperative assessment of patients who are at high risk of upgrading.

The limitations of our study are its retrospective character and relatively small number of subjects with GG1 (GS 3 + 3) and GG2 (GS 3 + 4) prostate cancer both at biopsy and after radical prostatectomy, which limits the generalizability of the results to a certain extent. However, it reflects a highly selected group of patients who are referred for prostatectomies in our institution, which as a consequence may increase the selection bias.

Due to the limited sample sizes within each Grade Group subgroup and the inherent correlation among diffusion parameters, multivariable analysis was not performed in the current study. Future studies with larger, multicenter cohorts are warranted to determine whether quantitative diffusion parameters serve as independent predictors of upgrading through multivariable modeling.

TRUS-guided systematic biopsies with 12 cores were performed and histopathologically evaluated both in our institution or outside our institution, which reflected the real-life situation in those years when patients were referred from different centers to our reference center for further diagnostics and treatment. Further studies using fusion TRUS/MRI targeted biopsies versus prostatectomies are needed to enhance the reproducibility of the findings in the MRI-pathway era of prostate cancer diagnosis.

All mpMRI and prostatectomies, as well as whole mount histopathology specimens were performed and evaluated in our institutions.

The researcher, who obtained quantitative parameters on MRI, was aware of the histopathological findings after prostatectomy, which allowed for a precise correlation of PCa foci detected on MRI with the ISUP Grade Groups of each PCa foci on whole-mount histopathology specimens. In our study, quantitative measurements of diffusion parameters were obtained by one reader, which was the reason why we were unable to perform an interobserver variability analysis.

Additionally, it should be acknowledged that both Gleason grading and PI-RADS scoring are subject to interobserver variability, which inevitably introduces a degree of subjectivity into the assessment. Standardized training and adherence to established grading criteria can mitigate, but not entirely eliminate, this limitation. Quantitative diffusion parameters, being objectively measured, may offer a potential advantage in this regard; however, interobserver reproducibility of ROI placement also warrants evaluation in future studies.

Future research should address the limitations of the present study through larger, prospective, multicenter designs incorporating MRI-targeted biopsies and whole-mount histopathology as the reference standard. Advanced analytical frameworks, including radiomics, texture analysis, and machine learning applied to quantitative DWI maps, may further improve the accuracy of preoperative grade prediction. The integration of novel biomarkers, such as the Prostate Health Index (PHI), with quantitative imaging parameters represents another promising direction. Furthermore, standardization of DWI acquisition protocols across different MRI vendors and field strengths will be essential to enable the clinical translation and broad applicability of these findings.

## 5. Conclusions

Quantitative diffusion parameters derived from standard monoexponential (ADC) and kurtosis (K) diffusion models demonstrated significant predictive value for ISUP Grade Group upgrading from TRUS biopsy to radical prostatectomy in patients with biopsy-proven GG1 and GG2 prostate cancer. In GG1, kurtosis K showed the highest performance (AUC 0.846), while in GG2, ADC performed best (AUC 0.814). Clinical parameters (PSA, PSA density) did not reach statistical significance in any group. These findings suggest a potential role for quantitative DWI as a non-invasive adjunct tool in preoperative risk stratification, with particular relevance to the selection of patients for active surveillance. Further prospective studies using larger multicenter cohorts, MRI-targeted biopsies, and advanced analytical approaches including radiomics and machine learning are needed to confirm and extend these findings.

## Figures and Tables

**Table 1:** Characteristics of study population, PI-RADS score and ISUP Grade Groups obtained in biopsy and after prostatectomy.

Parameter	Value
Number of patients	128
Age (years) mean	66
range	51–80
Prostate-Specific Antigen level (ng/mL) mean	9.67
range	2.2–28.5
Number of tumors studied	142
PI-RADS 3	4
PI-RADS 4	71
PI-RADS 5	67
ISUP Grade Group at biopsy (bGG)	
bGG 1	27
bGG 2	67
bGG 3	35
bGG 4	13
Tumor location of mpMRI lesions	
Tumor location N (%) Peripheral Zone	105 (74%)
Transitional Zone	37 (26%)
ISUP Grade Group at radical prostatectmy (pGG)	
pGG 1	9
pGG 2	47
pGG 3	61
pGG 4	13
pGG 5	12

**Table 2:** Mean values of diffusion parameters (D, K, ADC) and PSA in the upgrade and non-upgrade cohorts within GG 1 at biopsy.

	No Upgrade N (%)	Mean	SD	Upgrade N (%)	Mean	SD	*p*	*p*-Adj	Cohen’s d
**K**	7 (25.93%)	1.086	0.089	20 (74.07%)	1.262	0.146	0.001	0.005	1.355
**D**	7 (25.93%)	1.147	0.204	20 (74.07%)	0.97	0.101	0.063	0.189	−1.430
**ADC**	7 (25.93%)	0.891	0.134	20 (74.07%)	0.761	0.095	0.044	0.176	−1.397
**PSA**	6 (23.08%)	9.335	3.995	20 (76.92%)	7.521	3.658	0.35	0.420	−0.091
**PSAD**	6 (23.08%)	0.282	0.162	20 (76.92%)	0.185	0.075	0.21	0.420	−0.573

**Table 3:** Diagnostic performance of diffusion parameters and clinical variables in prediction of GG upgrade from biopsy to radical prostatectomy within GG 1 at biopsy.

	Threshold	AUC	95% CI lo	95% CI up	Sens	Spec
**K**	1.11	0.846	0.668	1	0.667	0.95
**D**	1.24	0.708	0.381	1	0.500	1.00
**ADC**	0.90	0.762	0.482	1	0.667	0.95
**PSA**	8.50	0.708	0.404	1	0.833	0.80
**PSAD**	0.25	0.750	0.467	1	0.833	0.85

**Table 4:** Mean values of diffusion parameters and clinical parameters in the upgrade and non-upgrade cohorts within GG 2 at biopsy.

	No Upgrade N (%)	Mean	SD	Upgrade N (%)	Mean	SD	*p*	*p*-Adj	Cohen’s d
**K**	31 (46.27%)	1.152	0.125	36 (53.73%)	1.311	0.188	<0.001	<0.001	0.761
**D**	31 (46.27%)	1.115	0.143	36 (53.73%)	0.914	0.175	<0.001	<0.001	−1.048
**ADC**	31 (46.27%)	0.862	0.111	36 (53.73%)	0.712	0.129	<0.001	<0.001	−1.079
**PSA**	29 (44.62%)	8.703	4.069	36 (55.38%)	10.216	5.492	0.207	0.414	0.354
**PSAD**	29 (44.62%)	0.255	0.139	36 (55.38%)	0.284	0.175	0.464	0.464	0.285

**Table 5:** Diagnostic performance for diffusion parameters and clinical variables in prediction of GG upgrade from biopsy to radical prostatectomy within GG 2 at biopsy.

	Threshold	AUC	95% CI lo	95% CI up	Sens	Spec
**K**	1.240	0.755	0.638	0.871	0.724	0.694
**D**	0.970	0.810	0.707	0.913	0.828	0.667
**ADC**	0.810	0.814	0.712	0.917	0.690	0.806
**PSA**	6.140	0.561	0.419	0.703	0.310	0.833
**PSAD**	0.217	0.557	0.408	0.706	0.586	0.667

**Table 6:** Mean values of diffusion parameters and clinical parameters in the upgrade and non-upgrade cohorts within GG 3 at biopsy.

	No Upgrade N (%)	Mean	SD	Upgrade N (%)	Mean	SD	*p*	*p*-Adj	Cohen’s d
**K**	27 (77.14%)	1.266	0.158	8 (22.86%)	1.342	0.129	0.181	0.905	0.459
**D**	27 (77.14%)	0.961	0.178	8 (22.86%)	0.913	0.197	0.544	1.000	−0.177
**ADC**	27 (77.14%)	0.753	0.129	8 (22.86%)	0.698	0.098	0.212	0.905	−0.346
**PSA**	27 (77.14%)	10.384	5.405	8 (22.86%)	9.862	2.494	0.705	1.000	−0.325
**PSAD**	27 (77.14%)	0.289	0.16	8 (22.86%)	0.329	0.12	0.464	0.905	0.144

**Table 7:** Mean values of diffusion parameters and clinical parameters in the upgrade and non-upgrade cohorts within GG 4 at biopsy.

	No Upgrade N (%)	Mean	SD	Upgrade N (%)	Mean	SD	*p*	*p*-Adj	Cohen’s d
**K**	9 (69.23%)	1.322	0.211	4 (30.77%)	1.455	0.246	0.391	1.000	0.600
**D**	9 (69.23%)	0.921	0.223	4 (30.77%)	0.833	0.225	0.537	1.000	−0.396
**ADC**	9 (69.23%)	0.704	0.144	4 (30.77%)	0.635	0.135	0.432	1.000	−0.491
**PSA**	9 (69.23%)	6.327	2.121	4 (30.77%)	10.385	5.725	0.253	1.000	1.161
**PSAD**	9 (69.23%)	0.199	0.122	4 (30.77%)	0.453	0.265	0.149	0.745	1.467

## Data Availability

The detailed data presented in this study are available from the corresponding authors upon request.

## Abbreviations

The following abbreviations are used in this manuscript:

ADC	Apparent Diffusion Coefficient
AS	Active Surveillance
AUC	Area Under the Curve
Dapp	Apparent Diffusion Coefficient corrected for non-Gaussian behavior
DKI	Diffusion Kurtosis Imaging
DWI	Diffusion-Weighted Imaging
GG	Gleason Grade Group
GS	Gleason Score
ISUP	International Society of Urological Pathology
K	Kurtosis
mpMRI	Multiparametric Magnetic Resonance Imaging
MRI	Magnetic Resonance Imaging
PCa	Prostate Cancer
PHI	Prostate Health Index
PI-RADS	Prostate Imaging—Reporting and Data System
PSA	Prostate-Specific Antigen
PSAD	Prostate-Specific Antigen Density
ROC	Receiver Operating Characteristic
ROI	Region of Interest
RP	Radical Prostatectomy
TLR	Toll-Like Receptor
TRUS	Transrectal Ultrasound
